# Facile Fabrication of Sandwich Structural Membrane With a Hydrogel Nanofibrous Mat as Inner Layer for Wound Dressing Application

**DOI:** 10.3389/fchem.2018.00490

**Published:** 2018-10-16

**Authors:** Xueqian Yin, Ya Wen, Yajing Li, Pengqing Liu, Zhongming Li, Yidong Shi, Jianwu Lan, Ronghui Guo, Lin Tan

**Affiliations:** ^1^College of Light Industry, Textile and Food Engineering, Sichuan University, Chengdu, China; ^2^College of Architecture & Environment, Sichuan University, Chengdu, China; ^3^College of Polymer Science and Engineering, Sichuan University, Chengdu, China

**Keywords:** sandwich structure, hydrogel nanofibrous mat, antibacterial activity, antioxidant activity, wound dressing application

## Abstract

A common problem existing in wound dressing is to integrate the properties of against water erosion while maintaining a high water-uptake capacity. To tackle this issue, we imbedded one layer of hydrogel nanofibrous mat into two hydrophobic nanofibrous mats, thereafter, the sandwich structural membrane (SSM) was obtained. Particularly, SSM is composed of three individual nanofibrous layers which were fabricated through sequential electrospinning technology, including two polyurethane/antibacterial agent layers, and one middle gelatin/rutin layer. The obtained SSM is characterized in terms of morphology, component, mechanical, and functional performance. In addition to the satisfactory antibacterial activity against *Staphylococcus aureus* and *Escherichia coli*, and antioxidant property upon scavenging DPPH free radicals, the obtained SSM also shows a desirable thermally regulated water vapor transmission rate. More importantly, such SSM can be mechanically stable and keep its intact morphology without appearance damage while showing a high water-absorption ratio. Therefore, the prepared sandwich structural membrane with hydrogel nanofibrous mat as inner layer can be expected as a novel wound dressing.

## Introduction

Hydrogel-based wound dressings are effective materials for the treatment of chronic wound due to the excellent exudates absorption and retaining properties, which allow them to modulate the fluid balance at the site of wound bed and maintain an microenvironment close to that present in native tissue (Fan et al., 2014; Sun et al., 2017; Zhao et al., 2017). Currently, most hydrogels are synthesized in terms of film, powder, particle and bulk 3D constructs. However, some drawbacks existing in those types of hydrogels when applying them as wound dressing materials, such as low gas and liquid permeation, insufficient surface area, poor structural integration (Ravichandran et al., 2014; Chen et al., 2015; Sun et al., 2017). Recently, hydrogel nanofibrous mats which can combine the advantages of both nanofibers and hydrogels have received increasing attention due to their desirable gas/liquid permeability, high surface area, highly porous structure, and excellent water uptake capacity(Brunelle et al., 2017; Sun et al., 2017; Zhao et al., 2017).

Electrospinning is an efficient and versatile technique to produce diversified nanofibers, and hundreds of polymers and their composites have been successfully processed into fibrous structure through electrospinning (Lin et al., 2012; Valizadeh and Mussa, 2014; Ahmed et al., 2015). Recently, several electrospun hydrogel nanofibrous mats, e.g., poly(vinylalcohol) (Bhowmick and Koul, 2016; Oh et al., 2016), Poly-(N-vinyl-2-pyrrolidone) (Kurniawan et al., 2016), poly(acrylic acid)-silica (Wang et al., 2016), poly(N-isopropylacrylamide) (Wen et al., 2015; Yao et al., 2015) have been reported, and the obtained hydrogel nanofibrous mats were mostly proposed to be applied as wound dressing and tissue scaffold. Nevertheless, the maintenance of intact hydrogel nanofibrous structure should be further improved during the functions performing due to the intrinsic poor mechanical stability (Fan et al., 2014; Gonzalez et al., 2014; Wang et al., 2015; Wu et al., 2017), thus additional bottom and top protection barriers for hydrogel nanofibrous layer are necessary (Xia et al., 2015; Kim et al., 2017). Regarding the wound dressing application, susceptible to water erosion and bacterial infection are fatal for wound healing, the membranes possessing two hydrophobic surfaces and one inner hydrophilic layer have greatly promising prospects in wound dressing application accordingly, while each layer can have and perform its own function separately, such as antibacterial property, high water uptake capacity, water resistance against erosion (Fan et al., 2014; Xu et al., 2015; Zhao et al., 2017). In other words, sandwich structure may be an ideal candidate for wound dressing fabrication. However, the conventional electrospun nanofibrous membrane is single layer or double layers. Obviously, the former type has the same wettability for its both surfaces, and the problem of relatively low adhesive force between two individual layers exists in latter type (Tan et al., 2015c; Trinca et al., 2017). Therefore, the fabrication of sandwich structural membrane with hydrogel nanofibrous mat may address the above concerns.

Both electrospun polyurethane and gelatin are popular materials for wound dressings application (Li et al., 2016; Resmi et al., 2016; Sahraro et al., 2016; Nian et al., 2018). Polyurethane (PU) currently is one of the most widely applied synthetic polymers. Compared with conventional polymers, PU possesses comprehensive excellent performances on following aspects, including hydrophobic property, easy preparation, recyclability, and good mechanical features, such as the stretching capability and elasticity (Janik and Marzec, 2015; Kucinska-Lipka et al., 2015); Gelatin (GE) is a natural protein which is derived from the partial and irreversible hydrolysis of collagen, and it also has been extensively employed as a hydrogel polymer (Zhao et al., 2015, 2018), but the poor intrinsic mechanical property of gelatin hinders its application, and the primary way to exert the functions of gelatin is to combine with other polymers (Tan et al., 2015c; Feng et al., 2016; Trinca et al., 2017).

Herein, we put forward the concept of fabricating one novel sandwich structural membrane (SSM) consisting of two hydrophobic electrospun PU layers as bottom and top surfaces, respectively, and one hydrophilic inner layer of gelatin hydrogel nanofibrous mat. Additionally, in order to accelerate the potential wound healing process, both antibacterial activity and free radicals scavenging capacity are introduced into such SSM. We demonstrate that the facilely fabricated SSM shows good performance on antibacterial activity, antioxidant property, water vapor transmission rate, mechanical and water uptake capacity. Therefore, the fabricated SSM in this study can be highly recommended as an ideal cover dressing for promoting wound healing.

## Materials and methods

### Materials

Polyurethane and antibacterial agent N-halamine (N-chloro-2,2,6,6-tetramethyl-4-piperidinyl, Ca) were synthesized according to the previous reports, and the synthesis information of Ca is presented in Figure S1 (Tan et al., 2015a; Dong et al., 2017). Rutin was purchased from Shanghai Aladdin Biochemical Polytron Technologies Inc.; Gelatin (Type A) from porcine skin was purchased from Sigma Aldrich. N, N-dimethylacetamide (DMAc) and other solvents were purchased from Chengdu Kelong reagent company and used directly without further purification. Dehydrated polycaprolactone diol (PCL, Mn = 4,000 g/mol) was obtained from Perstop U.K. Ltd (United Kindom). 4,4-methylene bis(phenylisocyanate) (MDI), 1,4-Butanediol (BDO) and dibutyltin dilaurate (DBTDL) were purchased from Sigma Aldrich (Unnithan et al., 2015).

### Synthesis of polyurethane

The applied polyurethane used for electrospinning in this study was synthesized according to our previous method. In particular, the obtained PU composed of PCL, MDI, and chain extender BDO. The reaction to prepare prepolymer was conducted in a 1,000 mL conical flask equipped with a mechanical stirrer. PCL (~492 g) mixed with MDI (~24 g) for 2 h at 80°C, and followed by the chain extension reaction with BDO (~100 g) for another 2 h under same temperature. The whole isocyanate group content was excessive by 3 mol%, and the hard segment content is around 20%. All the chemicals used in the synthesis process should be dehydrated with 4 Å molecular sieves in advance (Tan et al., 2015a).

### Preparation of gelatin-rutin (GE-R) and polyurethane-antibacterial agent (PU-Ca) solution

Firstly, 24 wt% gelatin solution was prepared by dissolving gelatin powder into a binary solvent consisting of water and formic acid with the mass ratio of 8:2 accordingly and the homogenous solution was obtained after stirring at room temperature for 3 h; Thereafter, a calculated amount of rutin powder (10 wt% of gelatin) was added to the solution for another 2 h stirring under the same condition. Similarly, PU-Ca dissolved in DMAc was prepared under constant stirring at 80°C for 4 h, the concentration of PU was fixed at 20 wt%, and the ratio of Ca was 10 wt% of PU content.

### Fabrication of electrospun sandwich structural membrane (SSM)

SSM was prepared using a manual assembly electrospinning system. The bottom and top layers were fabricated by spinning PU-Ca solution, and the inner hydrogel nanofibrous layer was made from GE-R solution, two voltage gradients of around 1.0 and 1.8 kV/cm were applied for PU-Ca solution and GE-R solution, respectively, and the feeding rate of 1 mL/h was fixed during electrospinning process under an ambient condition (Relative humidity = 50 ± 5%, Working temperature = 22~26°C). In the course of spinning process, we controlled the thickness of the electrospun membrane through feeding ratio, spinning time and the width for nanofiber collection, and the thickness was measured with the aid of a micrometer (Mitutoyo, Japan). The PU-Ca/GE-R/PU-Ca sandwich structure was constructed through sequential electrospinning. In our study, two SSMs were prepared by adjusting the thickness ratio among the three layers, particularly including 1:4:1 and 2:2:2, and we named such two SSMs in short as “SSM141” and “SSM222,” respectively. In addition, the PU-Ca and GE-R nanofibrous mats were prepared as comparisons. SSM141, SSM222, and GE-R nanofibrous mats were crosslinked by saturated glutaraldehyde vapor for 15 min and followed by placing in a vacuum desiccator to remove residual crosslinker.

### Characterizations

The surface and cross-section morphologies of nanofibrous mats were characterized by scanning electron microscope (SEM, Hitachi SU3500, Japan), and all specimens were sputter-coated with gold for 20 s before observation. Nanofiber diameter distribution was determined by measuring the diameters of 100 randomly selected nanofibers in SEM images with the aid of *ImageJ* software; The chemical structure and conformation of nanofibrous mats were analyzed by attenuated total reflectance model based Fourier transform infrared (ATR-FTIR, Tracer-100, Japan) spectroscopy in the range of 650–4,000 cm^−1^ at room temperature; Thermal decomposition behavior was investigated by thermogravimetric analysis (DTG-60, Shimadzu, Japan) with a heating scan from 30 to 800°C, and the flow rate of nitrogen gas was fixed at 50 mL/min; Mechanical properties was investigated using an electronic single yarn strength meter (YM061,China). The width of specimens employed was 3 mm and the distance between two clamps was 20 mm, the stretching speed was 10 mm/min, and each sample was measured at least 10 times, the average values of breaking force (cN) and elongation at break (%) were recorded; Water contact angle (WCA) of the fabricated membranes surface was measured through a contact angle tester (Harke-SPCAX1, China) using the sessile drop method, and at least five individual values were collected and averaged.

### Water-absorption ratio and water vapor transmission rate (WVTR) investigation

WAR was evaluated by immersing samples in PBS buffer (pH = 7.4) for 12 h, and then weighted them after removing the surface water using wiping paper; WVTR was determined based on evaporation of water vapor through the testing nanofibrous mats under the temperature of 21 and 37°C with a continuous % RH, including 35, 55, and 75%. WVTR was monitored by measuring the weight loss (mg) of the cups by functions of unit area (cm^2^) and time (h), the cups without samples were applied as comparisons. Tests of both WAR and WVTR were performed in triplicate.

### Antibacterial activity assessment

Antibacterial activity of SSM against *Staphylococcus aureus* and *Escherichia coli* was investigated based on agar disk diffusion susceptibility. The samples were cut into discs with the size of 1.5^*^1.5 cm^2^, and sterilized by ultraviolet irradiation 15 min for both surfaces separately in a laminar flow hood. Gram-negative *E. coli* and Gram positive *S. aureus* were selected as representative bacteria and were cultivated overnight in an incubator, and the sterilized samples were placed on the surface of agar plates coated with the *E. coli* and *S. aureus* already diluted by PBS buffer. Subsequently, the plates were incubated at 37°C overnight, and then inhibition zones were observed and photographed.

### DPPH radical scavenging activity

The antioxidant activity of SSM were determined according to 1,1-diphenyl-2-picrylhydrazyl (DPPH) free radical-scavenging assays. Specifically, a piece of nanofibrous mat with the size of 1.5^*^1.5 cm^2^ was immersed in 4 mL DPPH ethanol solution (1.5 mg/mL), scavenging activity assay was carried out by recording the absorbance at 516 nm of DPPH solution at six specific time points, including 2, 4, 8, 16, 32, and 64 min after the mixture reaction, and the lower average absorbance value indicates better antioxidant activity.

## Results and discussion

### Morphological analysis

**Figure 1:**
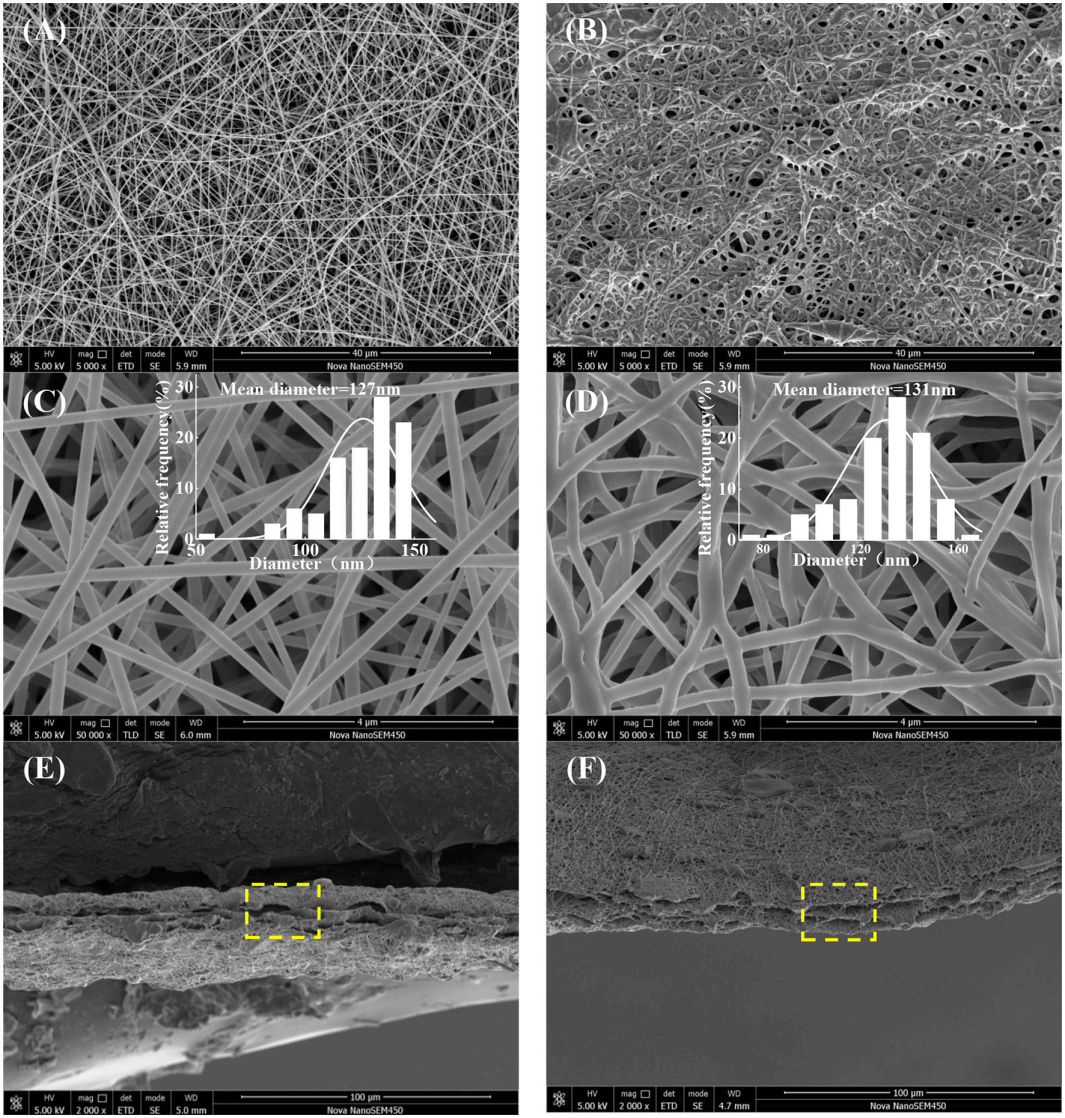
Surface images and fibers diameter distribution (*ImageJ*) of GE-R **(A,C)**, and PU-Ca **(B,D)**, and cross-sectional images of SSM141 **(E)**, and SSM222 **(F)**.

SEM images of surface and cross-section of electrospun nanofibrous membranes are presented in Figure 1. Firstly, all the as-fabricated nanofibrous mats show desirable fiber morphology and size homogeneity without beads (Figures 1A–D), while slow evaporation of DMAc, bending deformation, entanglement of the nanofibers and resulted in the compacted homogenous structure (Figure 1B), and the average diameter of GE-R and PU-Ca are 127 and 131 nm, respectively. Secondly, the obvious sandwich structure can be observed, and the distinct boundary exists between the adjacent two layers, while interconnected porous structure due to the entanglement of nanofibers are visible from the cross-sectional sites (Figures 1E,F). Unlike the conventional bilayered nanofibrous mats, such kind of sandwich structure can ensure the integration of nanofibrous membrane during the application, in other words, under the protection of bottom and top layers which are hydrophobic and materials-homogenous, such SSM can prevent the mutual stripping, collapse and effusion under the circumstance of water erosion or others. Additionally, the highly porous structure is highly beneficial to the circulation of oxygen gas and water vapor. More importantly, the wound exudates can freely access to the inner hydrogel nanofibrous layer through the porous pathway and stay inside the layer up to the saturated absorption.

### FT-IR characterization and thermal property investigation

**Figure 2:**
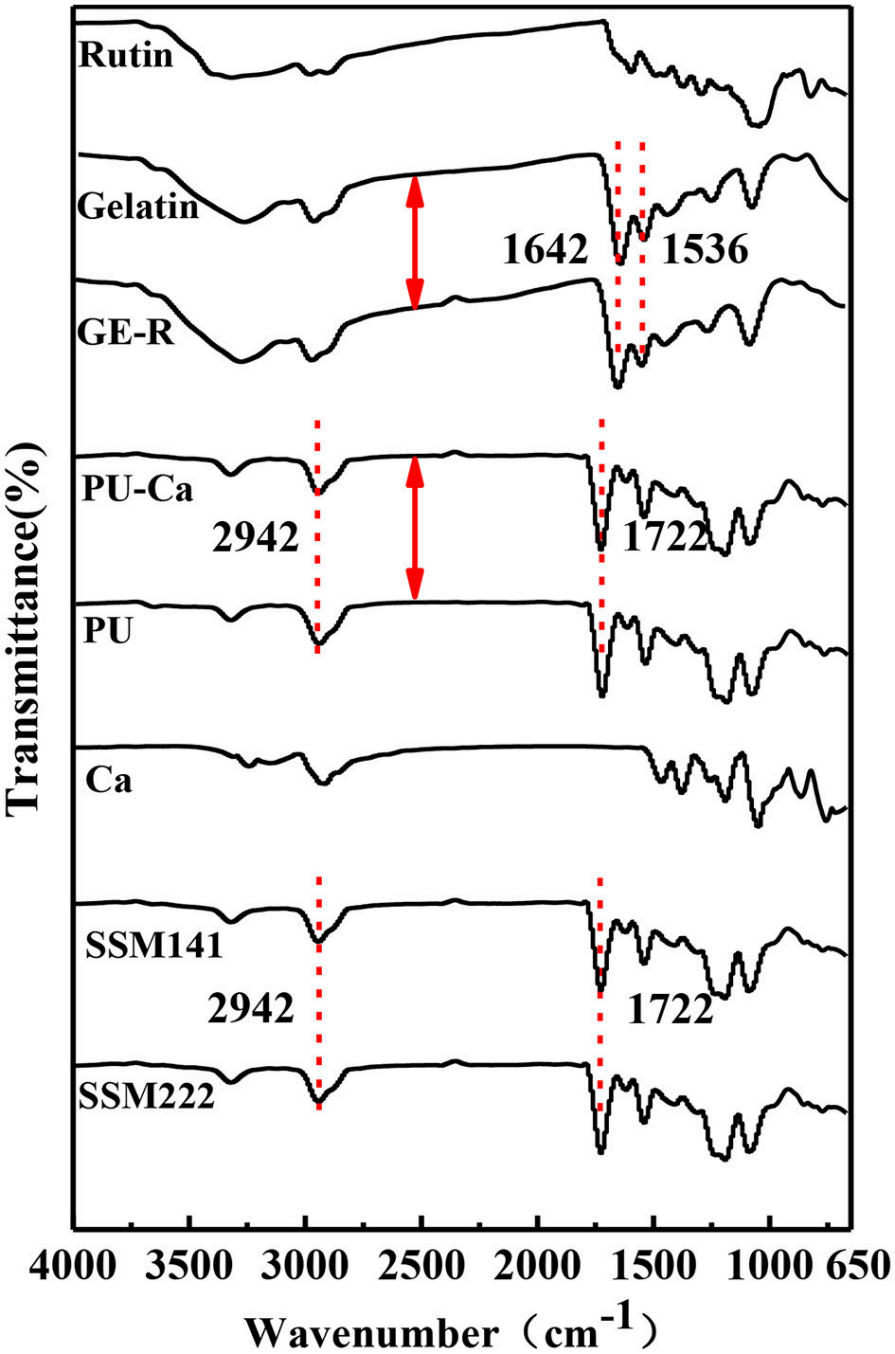
FT-IR spectrum of each component of SSM.

As shown in Figure 2, the typical strong peaks of relevant bonds indicate the blending and electrospinning process did not influence the structure of each component. Particularly, it can be clearly observed that the characteristic peak at ~1,642 cm^−1^ represents the C=O stretching vibration of peptide bonds in the backbone of gelatin, and the peak at ~1,536 cm^−1^ corresponds to the N-H bending and C-N stretching vibration; Additionally, the sharp peaks at ~1,722 cm^−1^ and broad peak at 2,942 cm^−1^ can be ascribed to C=O stretching vibration of urethane groups and stretching vibration of –NH and –OH groups of PU, respectively (Tan et al., 2015b). After they blended with rutin or antibacterial agent Ca, and even after the formation of SSM, the above typical peaks did not shift, thus, it can be concluded that all the components maintain their own native structure after the physical blending and electrospinning.

**Figure 3:**
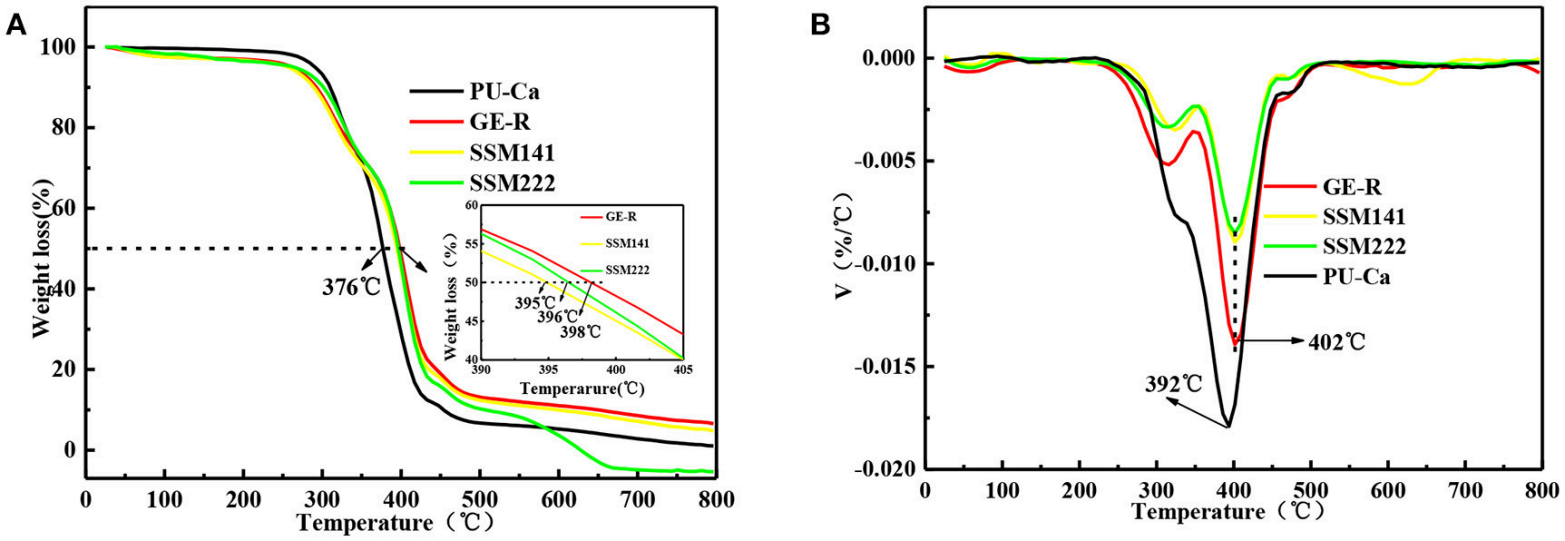
Thermal decomposition **(A)** and DTG **(B)** curves of different electrospun membranes.

The thermal decomposition behavior was also studied, and the decomposition curves are shown in Figure 3, Figures S2A,B. Regarding the semi-decomposition temperature (SDT) and maximum decomposition temperatures (MDT), 167, 374, 396°C and 181, 382, 408°C are recorded for Ca, PU-Ca, and PU, that means the thermal stability of PU decreases over 20°C after blending with Ca; In contrast, the stability of gelatin was significantly improved after blending with rutin in terms of SDT and MDT, e.g. the MDT of GE-R reach to 326°C, while only 276°C was recorded for the MDT of gelatin (Figures S2C,D). Additionally, from Figures 3A,B, both SSMs shows desirable thermal stability with near 400°C of SDT and above 400°C of MDT. In addition, there is no obvious difference between SSM141 and SSM222 in terms of SDT and MDT, indicating that the change of the thickness ratio of each layer has little influence on the thermal stability of the sandwich structural nanofibrous membranes, and such desirable thermal stability can endow the possible thermal sterilization of SSM before use.

### Mechanical properties

**Figure 4:**
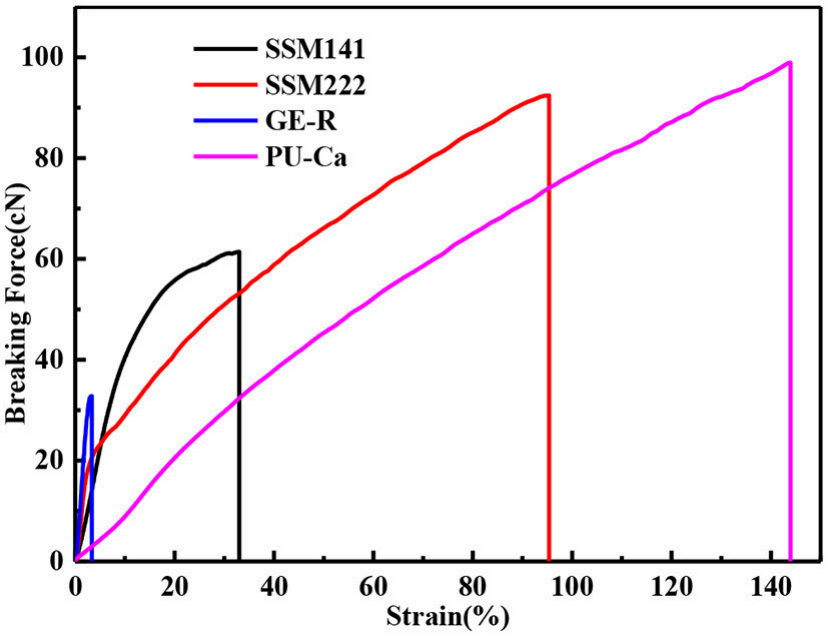
Stress-strain curves of different membranes.

A certain mechanical strength can cater to the motorial behavior of skin, while weak mechanical strength may cause the collapse of wound dressings and then fail to protect the wound bed. Figure 4 shows the representative stress-strain curves of different electrospun membranes. In this study, breaking force and elongation were recorded to evaluate the mechanical properties, and the data were summarized in Table 1. Obviously, GE-R has the lowest breaking force (~33 cN) as well as the breaking elongation (~4.0%), thus gelatin in combination with other polymers, e.g., PU, for wound dressing application is preferable. From the testing results, SSMs show much better mechanical property compared with that of GE-R electrospun membrane, the reason can be mainly attributed to the presence of PU-Ca layers, and higher composition ratio of PU-Ca endows SSM better mechanical property, i.e., SSM222 have desirable breaking force of around 96 cN and elongation of around 99%. Thus, the obtained SSM can withstand the mechanical deformation resulted from the motorial behavior.

**Table 1:** The average tensile strength, thickness, elongation at break of different membranes.

**Sample**	**Membrane thickness/μm**	**Breaking force/cN**	**Elongation at break/%**
GE-R	47.40 ± 0.89	32.66 ± 0.19	3.89 ± 0.88
SSM141	47.60 ± 1.58	60.36 ± 1.51	29.6 ± 4.67
SSM222	49.20 ± 1.22	95.57 ± 4.44	99.22 ± 6.05
PU-Ca	48.40 ± 1.58	98.67 ± 0.40	140.60 ± 4.57

### Surface wettability

**Figure 5:**
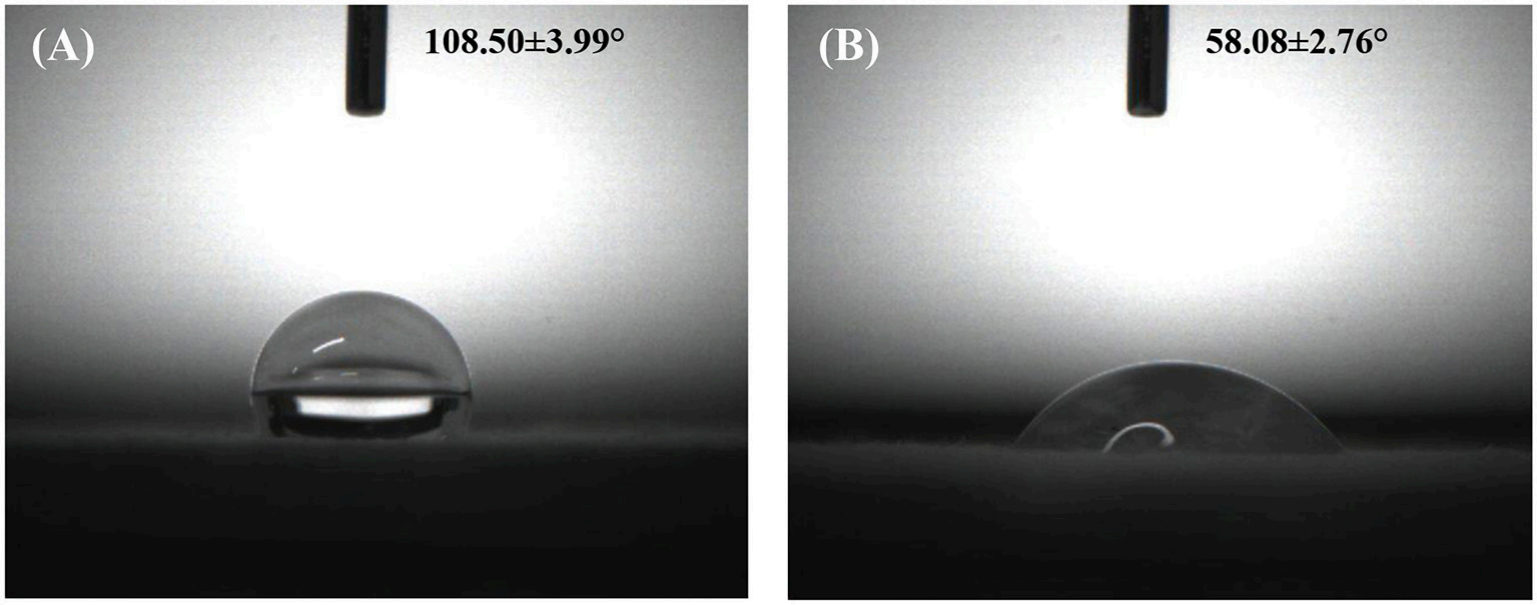
Water contact angle images of different membranes PU-Ca **(A)** and GE-R **(B)**.

Surface wettability is an important property for wound dressing which can influence not only the biological response but the defense performance against water erosion. Figure 5 shows the WCA values of two individual layer surfaces which consist the final SSMs, and the layer of PU-Ca is hydrophobic with a WCA value of 108.50 ± 3.99°, while the hydrogel nanofibrous layer of GE-R exhibits relative hydrophilic with a WCA value of 58.08 ± 0.76°. The WCA values of SSM141 and SSM222 were shown in Figure S3, and they have little difference with pristine PU-Ca layer, indicating that the inner layer of the hydrogel nanofiber has no significant effect on the surface wettability of SSM. Consequently, the bottom and top layers of the fabricated SSMs can effectively defense the water or wound fluids erosion and keep the structural integrity of SSMs during the application, and the inner hydrogel nanofibrous layer can absorb the wound fluids which can freely access through the nanofibrous pathway.

### WAR and WVTR study

Both WAR and WVTR are critical factors for the wound management, and both of them play their own role separately and synergistically. Particularly, WAR is applied to evaluate the exudates retaining capacity of the wound dressing, and WVTR will influence the wounds dehydration or accumulation of exudates. In virtue of desirable WAR and WVTR, an ideal moisture microenvironment will exist around the site of wound bed, thus promote the wound healing process.

WAR and WVTR are calculated according to following Equation (1) and Equation (2). In Equation (1), W_1_ represents the weight after the suction, and W_0_ represents the original weight of the sample.

(1) $$\text{WAR}(\%)=\frac{{\text{W}}_{1}-{\text{W}}_{0}}{{\text{W}}_{0}}\times 100\%$$

(2) $$\begin{array}{l} \text{WVTR}(\text{mg}.{\text{cm}}^{-2}.{\text{h}}^{-1})=\\ \frac{\text{Water weight change }(\text{mg})}{\text{Exposure area }({\text{cm}}^{2})\times \text{exposure time}(\text{h})} \end{array}$$

**Figure 6:**
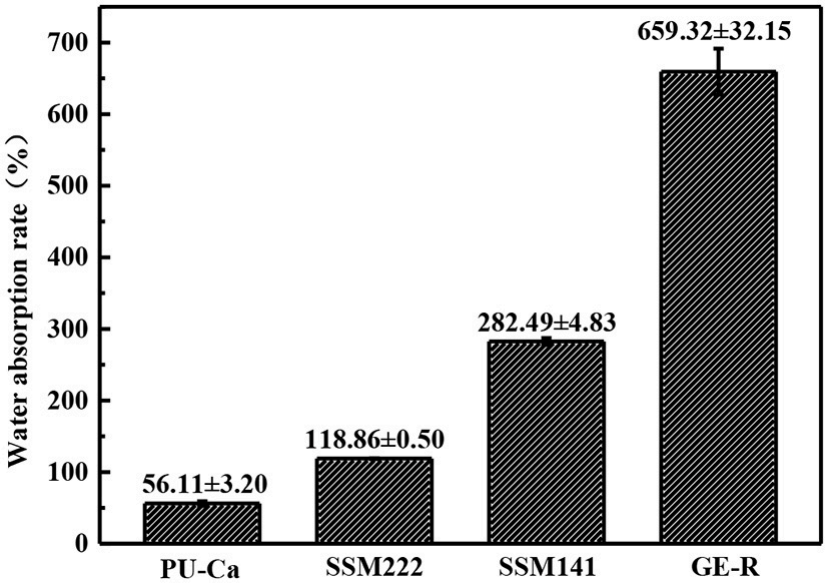
WAR of different electrospun membranes under ambient condition.

**Figure 7:**
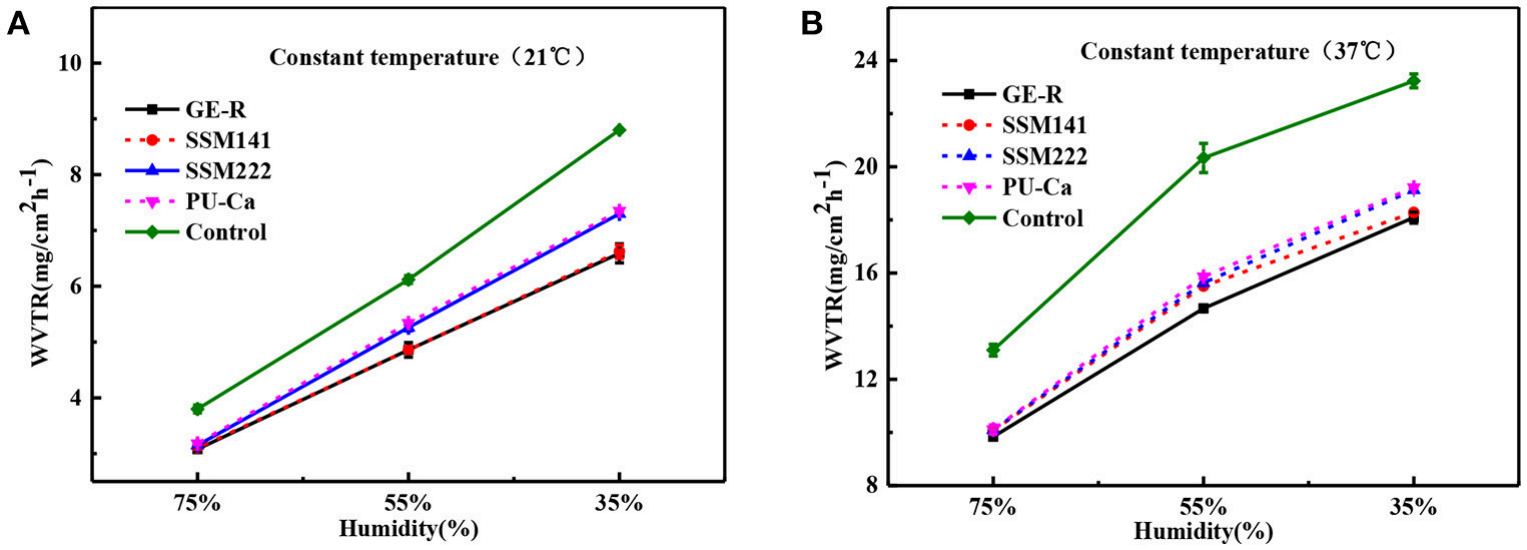
WVTR curves by the function of humidity under constant temperature 21°C **(A)** and constant temperature 37°C **(B)**.

The thickness of the obtained nanofibrous membranes are around 50 μm. As shown in Figure 6, the WAR values of PU-Ca, SSM222, SSM141, and GE-R are around 56, 119, 282, and 659%, respectively. The WAR of SSM222 and SSM141 increased by 2 and 5 times higher than that of PU-Ca membrane, which indicates the incorporation of hydrogel nanofibrous mat can significantly increase the WAR, and the thicker of GE-R layer incorporated in such sandwich structural membrane renders higher WAR owning to the intrinsic hygroscopicity of gelatin. Therefore, the accumulation of exudates around the wound beds can be avoided and the risk of potential bacteria infection will be reduced. Whatever normal skin or injured skin, body fluid will evaporate through the micropores of skin, while the injured skin normally shows higher WVTR of 1.16–21.41 mg.cm^−2^.h^−1^ compared to that of normal skin (Saeed et al., 2017). Thus, porous structure with suitable WVTR of wound dressing is essential and required. Figure 7 and Figure S4 show the successive recorded WVTR values of different electrospun membranes under different temperature and humidity. It can be clearly seen that the WVTR values for all electrospun membranes reasonably increase with temperature increasing under constant humidity, while WVTR decreases with increasing the ambient humidity. From Table 2, all WVTR values of SSMs, lowest value of 3.10 ± 0.02 mg/cm^2^.h^−1^ and the highest value of 19.13 ± 0.04 mg/cm^2^.h^−1^, are within the body fluids evaporation range of injured skin, which can indicate that the fabricated SSM can meet the requirement on managing the wound fluids as a qualified wound dressing.

**Table 2:** WVTR and WAR of different electrospun membranes.

**Samples**	**Thickness (μm)**	**WVTR/21**^**°**^**C (mg/cm**^**2**^**.h**^**−1**^**) Humidity(%)**			**WVTR/37**^**°**^**C (mg/cm**^**2**^**.h**^**−1**^**) Humidity(%)**			**WAR(%)**
		**35%**	**55%**	**75%**	**35%**	**55%**	**75%**	
GE-R	47.40 ± 0.89	6.59 ± 0.17	4.86 ± 0.13	3.08 ± 0.03	18.09 ± 0.21	14.67 ± 0.02	9.84 ± 0.07	659.32 ± 32.15
SSM141	47.60 ± 1.58	6.62 ± 0.12	4.86 ± 0.05	3.10 ± 0.02	18.28 ± 0.09	15.51 ± 0.09	10.08 ± 0.09	282.49 ± 2.83
SSM222	49.20 ± 1.22	7.30 ± 0.005	5.26 ± 0.06	3.15 ± 0.03	19.13 ± 0.04	15.63 ± 0.04	10.10 ± 0.15	118.8600.50
PU-Ca	48.60 ± 1.58	7.35 ± 0.03	5.34 ± 0.01	3.18 ± 0.02	19.22 ± 0.03	15.86 ± 0.04	10.11 ± 0.17	56.11 ± 3.20
Control	NA	8.80 ± 0.16	6.12 ± 0.07	3.8 ± 0.07	23.23 ± 0.26	20.33 ± 0.55	13.10 ± 0.22	NA

### Antioxidant property

Antioxidant property of the fabricated sandwich structural membrane was evaluated in term of the capacity on scavenging free radicals, and the scavenging ratio of DPPH free radicals (SRDPPH) was calculated according to the following Equation (3):

(3) $$\text{SRDPPH}(\%)=\frac{{\text{A}}_{0}-{\text{A}}_{\text{t}}}{{\text{A}}_{0}}\times 100\%$$

**Figure 8:**
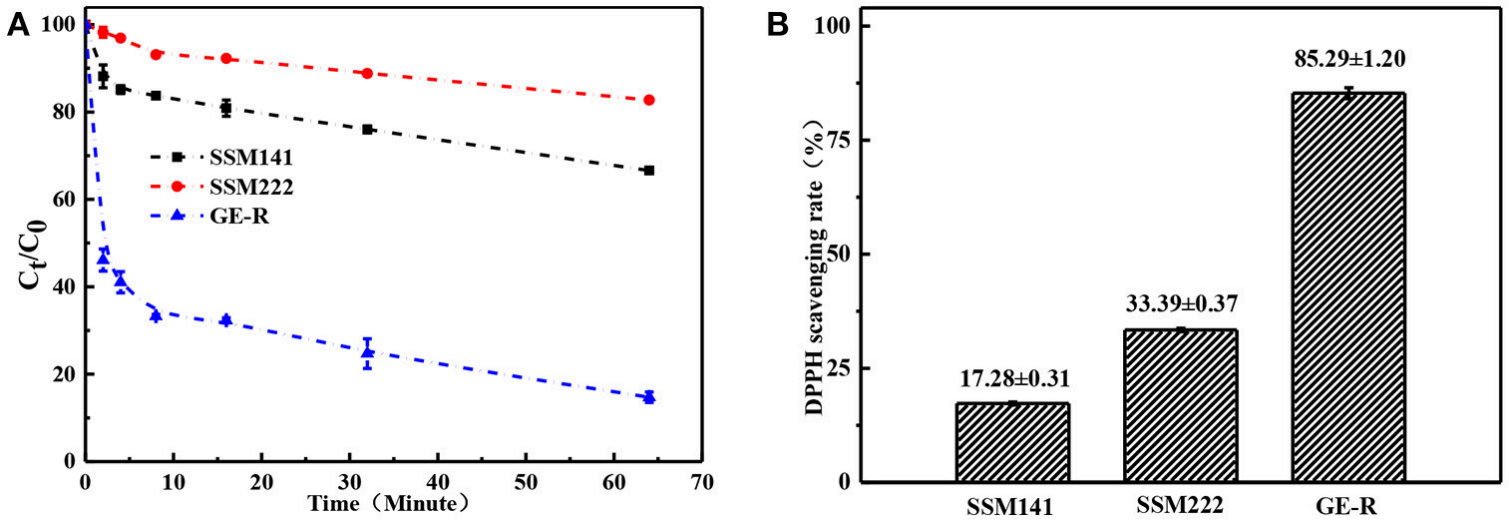
Antioxidant performance of different electrospun membranes. **(A)** Scavenging rate-time curve of different membranes; **(B)** Scavenging rate histogram of different membranes.

Where A_0_ is the original absorbance of DPPH, A_t_ is the absorbance of the DPPH at the specific measuring time. Antioxidant property refers to the ability of scavenging free radicals, and it should be a crucial property for wound dressing which can protect human skin or wound bed from free radicals damage. Antioxidant performance was monitored through measuring the absorbance of DPPH-contained solution at 516 nm, which was also accompanied by a color change from deep purple to yellow. Figure 8 demonstrates the antioxidant activity of SSM prepared with different contents of rutin, and higher content of rutin renders the higher SRDPPH, that means higher content of GE-R layer can be incorporated to prepare SSM with better antioxidant performance. In addition, the SRDPPH was recorded by the function of time (Figure 8B), and the slope of the curve can reflect the scavenging rate of DPPH free radicals. We can clearly observe that the oxidant performance of all the samples include one initial fast SRDPPH stage with a sharp slope and one slower stage. In other words, the electrospun membranes can initially scavenge the DPPH free radicals quickly then reach a plateau gradually.

### Antibacterial activity

**Figure 9:**
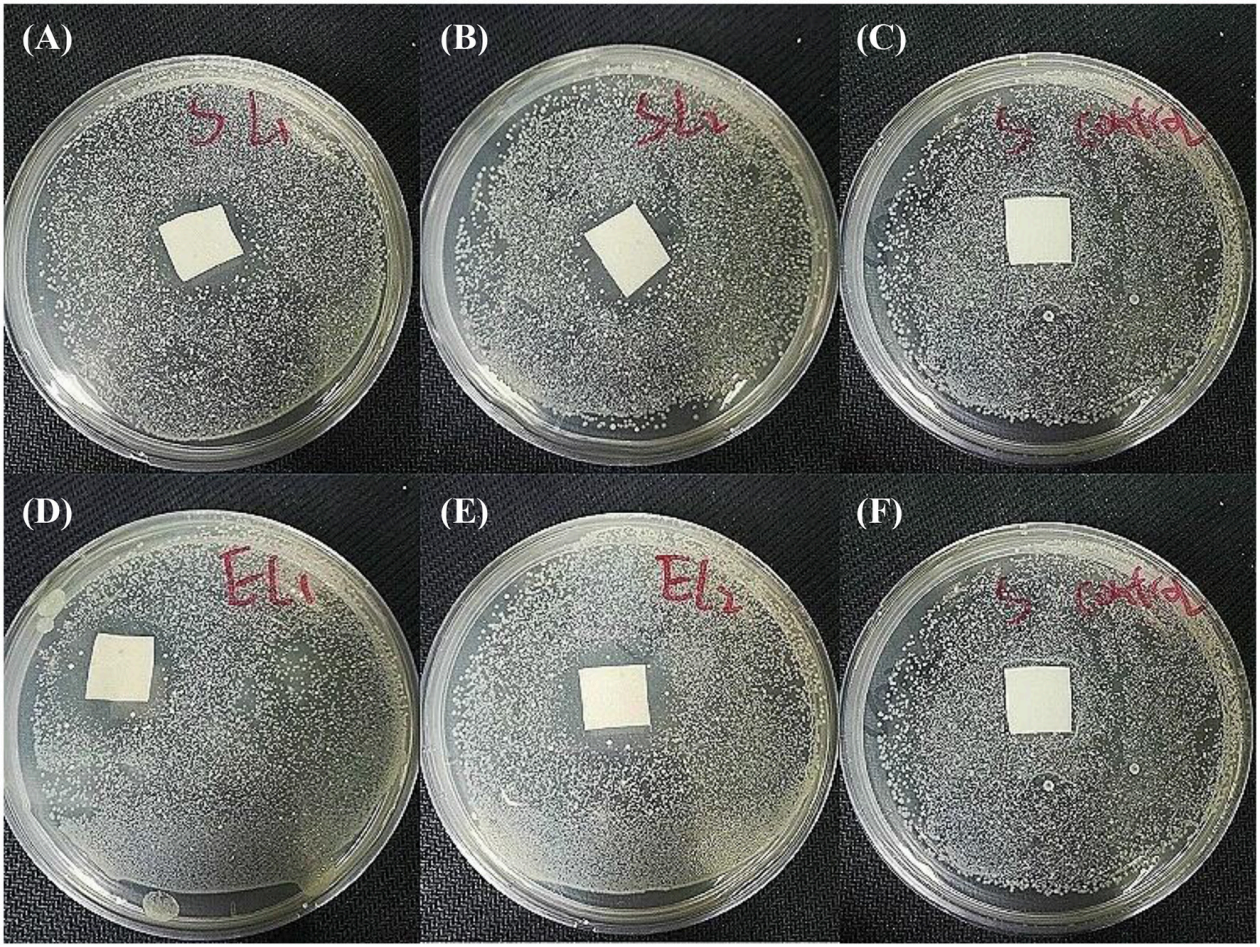
Antibacterial activity investigation results. SSM141against *S. aureus* **(A)** and *E. coli* **(D)**. SSM222 against *S. aureus* **(B)** and *E. coli* **(E)**, and pristine PU against *S. aureus* **(C)** and *E. coli* **(F)** as the comparisons.

Antibacterial activity is essential to a qualified wound dressing, that's because a longer healing process will be required once the wound bed is infected by bacteria. Figure 9 shows the antibacterial activity of two SSMs against *E. coli* and *S. aureus*. Compared with the pristine PU membrane, PU-Ca (Figure S5), SSM141 and SSM222 have satisfactory antibacterial activity by the evidence of obvious inhibition zones. In addition, we observed that the inhibition zones against *E. coli* is slightly larger than those against *S. aureus*, the possible reason can be attributed to the thicker cell wall of the *Gram*-positive negative bacteria, and thus the encapsulated antibacterial monomer N-halamine (Ca) can more effectively kill the *Gram*-negative bacteria.

## Conclusion

In this study, we have successfully fabricated a novel type of electrospun SSM with a hydrogel nanofibrous mat as the inner layer through lay-by-layer deposition. The obtained SSM has two hydrophobic surfaces (PU-Ca) which can prevent the water erosion, and simultaneously has a high water-uptake capacity because of the hydrophilic hydrogel nanofibrous layer (GE-R), thus the structural integrity can be effectively maintained during the application. Additionally, the SSM also displays favorable antibacterial activity against *E. coli* and *S. aureus*, antioxidant activity in terms of scavenging capacity of DPPH free radicals, and a desirable thermally regulated WVTR. More importantly, the functions and properties can be highly adjusted owing to the nanofibrous sandwich structure, and the incorporated functions can be also performed individually, such as the thickness of inner hydrogel nanofibrous layer, wettability of each layer, mechanical strength and antioxidant period. We believe that such type of SSM can work competently as a wound dressing, and more functional SSMs can be prepared based on this concept.

## Author contributions

XY, YW, and YL completed the experiments. PL and ZL provided some assistance on conducting experiments and testing. YS, JL, and RG gave some suggestions on writing. LT proposed the idea, designed the experiment, gave scientific support, revised, and finalized the manuscript.

### Conflict of interest statement

The authors declare that the research was conducted in the absence of any commercial or financial relationships that could be construed as a potential conflict of interest.
