# Supplementary material for: Facile Fabrication of Sandwich Structural Membrane With a Hydrogel Nanofibrous Mat as Inner Layer for Wound Dressing Application
Source: Front Chem. 2018 Oct 16;6:490. doi: 10.3389/fchem.2018.00490 (PMC6201043; doi:10.3389/fchem.2018.00490)
Supplement: Supplementary file 1 [file Table_1.doc]

Supporting Information

**Facile fabrication of** **sandwich structural membrane with a** **hydrogel nanofibrous mat as inner layer for wound dressing application**

**Xueqian Yin^1^, Ya Wen^1^, Yajing Li^1,3^, Yidong Shi^1^, Jianwu Lan****^1^, Ronghui Guo^1^, Pengqing Liu^2^, Zhongming Li^2^, Lin Tan^1,2^***

^1^College of Light Industry, Textile and Food Engineering, Sichuan University, Cheng Du 610065, China

^2^College of Polymer Science and Engineering, Sichuan University, Chengdu 610065, Sichuan, China

^3^College of Architecture & Environment, Sichuan University, Cheng Du 610065, China

*** Correspondence:**Dr. Lin Tan, [tanlinou@scu.edu.cn](mailto:tanlinou@scu.edu.cn)


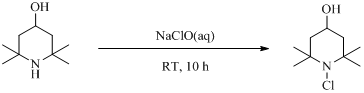


**Fig. S1 丨**The synthesis of antibacterial monomer Ca .

| **(A)**  **(B)** |  |
| --- | --- |
| **(C)**  **(D)** |  |

**Fig. S2丨**TG and DTG curves of all the materials that make up the composite membranes. (A)TG curves of PU-Ca, PU electrospun membranes and Ca powders; (B) DTG curves of PU-Ca, PU electrospun membranes and Ca powders; (C) TG curves of electrospun membranes and Rutin powders; (D) DTG curves of electrospun membranes and Rutin powders.

| 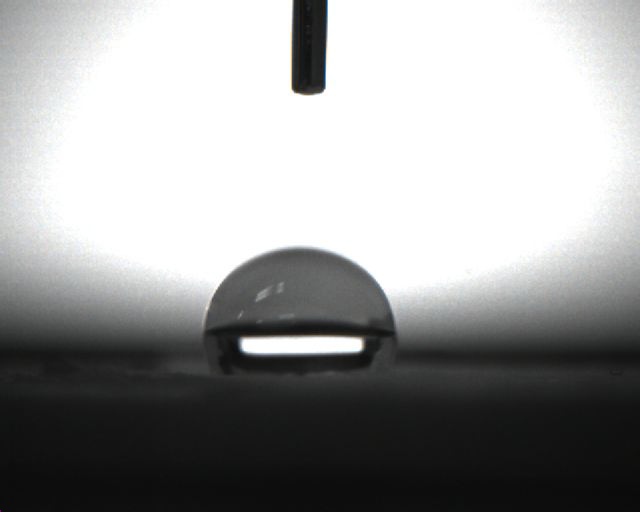  **(A)**  **107.50±2.33°** | 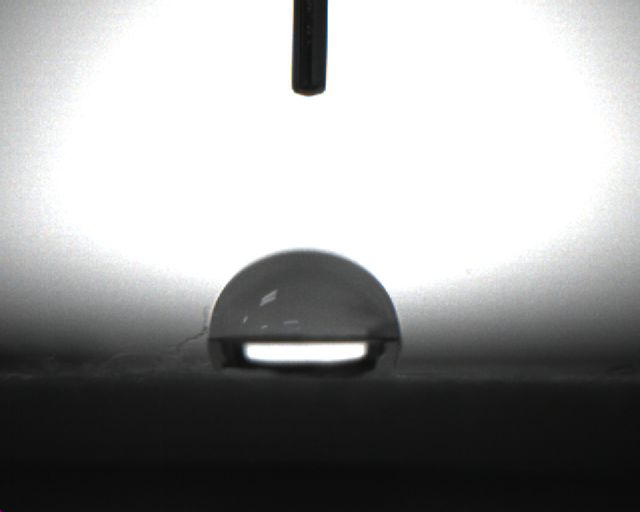  **(B)**  **110.05±3.51°** |
| --- | --- |

**Fig. S3 丨**Water contact angle images of different membranes (A) SM222, (B) SSM141.

**(A)**

**(B)**

|  |  |
| --- | --- |
| **(C)** | |

**Fig. S4**丨WVTR curves by the function of temperature under (A) constant humidity (35%), (B) constant humidity (55%) and (C) constant humidity (75%).

| **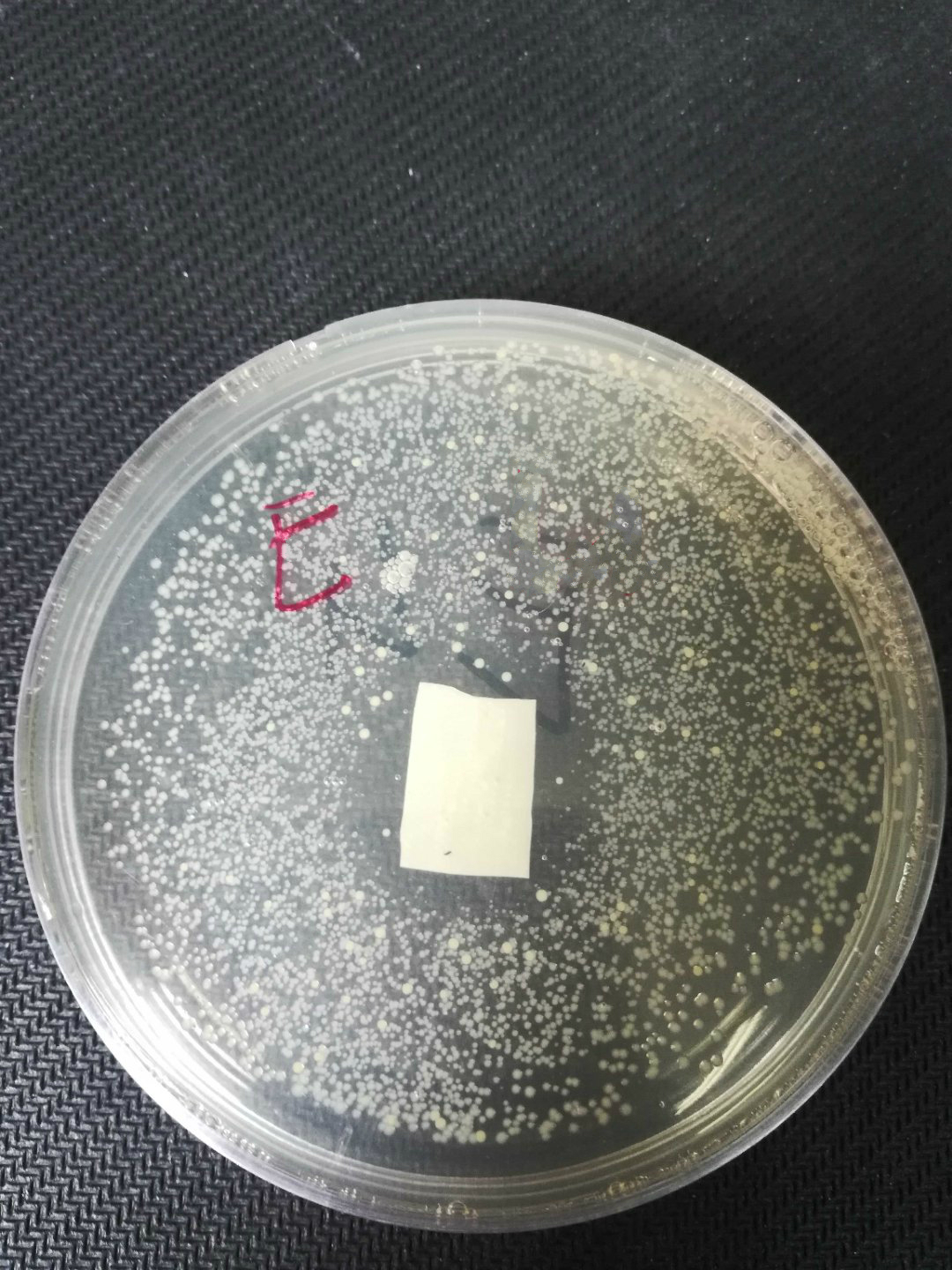**  **(A)** | **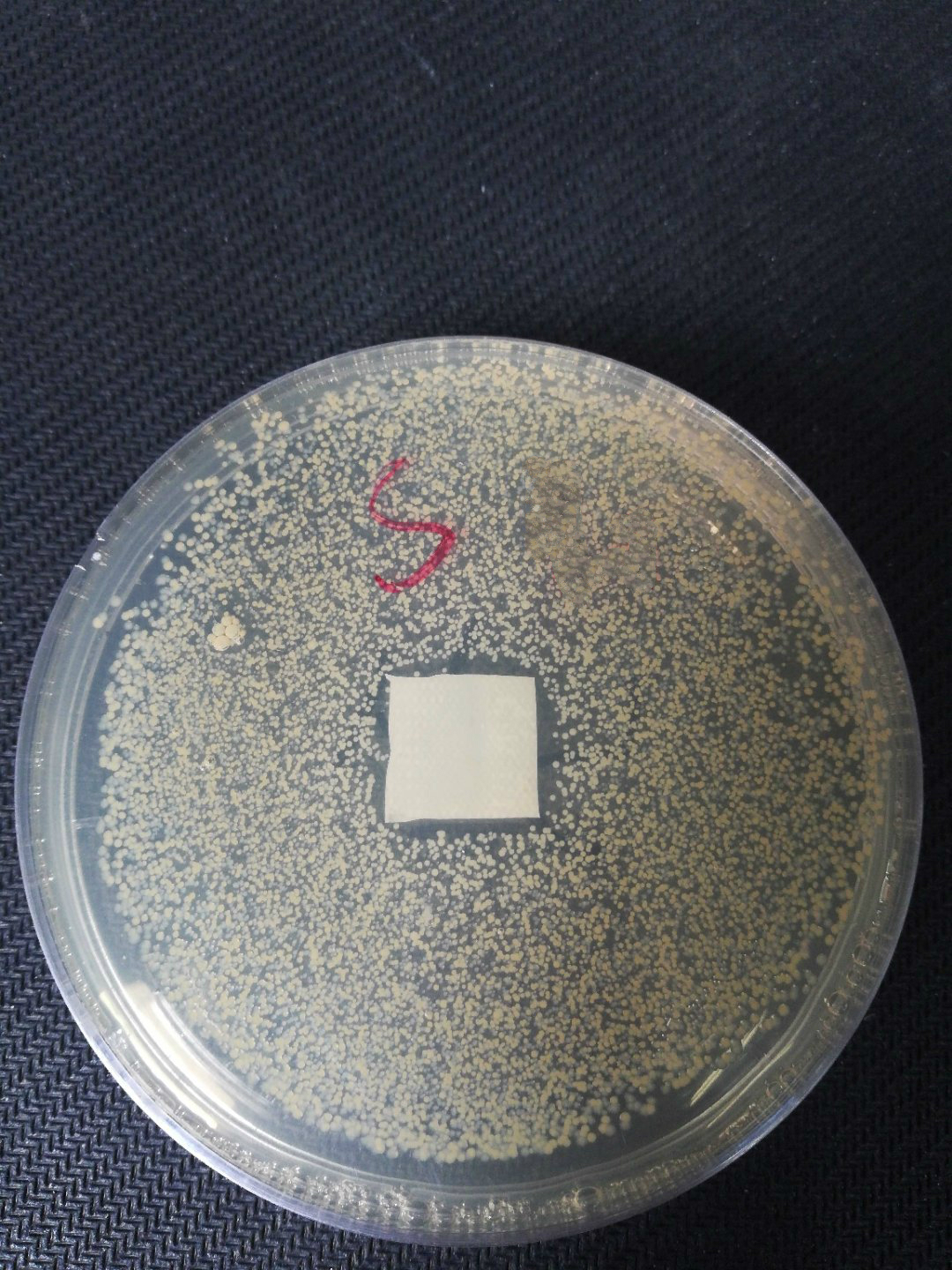**  **(B)** |
| --- | --- |

**Fig. S5 丨**Antibacterial activity of PU-Ca against *E. Coli* (A) and *S. Aureus* (B).

**Reference**

Dong, A., Wang, Y.J., Gao, Y., Gao, T., and Gao, G. (2017). Chemical Insights into Antibacterial N-Halamines. *Chemical Reviews* 117**,** 4806.
